# Factors Affecting Hypertension in the Adult Population of the Marmara Region, Turkey: A Descriptive Field Study

**DOI:** 10.1155/2020/8869042

**Published:** 2020-12-31

**Authors:** Olgun Göktaş, Tunay Şentürk, Canan Ersoy

**Affiliations:** ^1^Family Practice Unit, Bursa Uludağ University, Family Health Center, Bursa, Turkey; ^2^Department of Cardiology, Bursa Uludağ University, Faculty of Medicine, Bursa, Turkey; ^3^Department of Internal Medicine, Division of Endocrinology and Metabolism, Bursa Uludağ University, Faculty of Medicine, Bursa, Turkey

## Abstract

**Introduction:**

Hypertension is an increasingly prevalent global public health problem. Nutritional culture and lifestyle are among the factors related to hypertension. The aim of this study was to evaluate the prevalence and influential factors of hypertension in the adult population of the Marmara region, Turkey.

**Methods:**

The study was conducted in 10 provinces in the Marmara region between June 01, 2018, and November 30, 2018. Participants included 2353 patients over 18 years of age diagnosed with hypertension by any of the 30 family physicians working in the Family Health Centers in these provinces. After the participants provided written consent, a survey consisting of 25 questions was administered by their family physicians. SPSS 25.0 (IBM Corporation, Armonk, New York, United States) was used for all statistical analysis calculations.

**Results:**

The patients included 1449 females (61.6%) and 904 males (38.4%). Among the respondents, 1555 (73.1%) had primary hypertension etiology and 572 (26.9%) had secondary etiology. While 1614 patients (68.6%) did not exercise at all, 739 patients (31.4%) reported exercising; 1026 patients (43.9%) did not restrict salt in their diet; and 1134 patients (48.2%) had a family history of hypertension.

**Conclusion:**

Since individual and environmental factors affect the etiology of hypertension, it is recommended that family physicians address these factors first as part of a holistic approach for hypertension prevention, diagnosis, treatment, and follow-up.

## 1. Introduction

Hypertension is an increasingly common public health problem in developed and developing countries. It is also one of the most common causes of death in every region of the world [1, 2]. The Seventh Report of the Joint National Committee on Prevention, Detection, Evaluation, and Treatment of High Blood Pressure (BP) (JNC7) described hypertension as a systolic blood pressure (SBP) ≥140 mmHg and/or diastolic blood pressure (DBP) ≥90 mmHg [3]. These values were also accepted as the hypertension diagnostic thresholds in many different guidelines reported after JNC7 [4–6]. Worldwide, hypertension is especially relevant to cardiovascular and renal diseases, and the prevalence of the condition is gradually increasing [7, 8]. Several studies have examined the prevalence and factors influencing hypertension in different parts of the world [9–11], demonstrating cultural differences in the disease. There are cultural and regional differences in the rates of hypertension awareness, prevalence, affecting factors, results, and benefits.

Many national and internationally accepted guidelines from Europe and North America offer recommendations for diagnosing and treating hypertension. However, there are differences in these guidelines, and some suggestions are not consistent with clinical practice in Turkey [12] owing to the cultural differences in nutrition and lifestyle, such as the excessive use of salt. Such inconsistent guidelines can lead to increases in the prevalence of hypertension [13]. Awareness, treatment, and control of hypertension are especially important to the prevention and management of chronic kidney disease [14]. Considering the prevalence and seriousness of hypertension and the inconsistent information about this health problem in Turkey, we aimed to evaluate the prevalence of hypertension and its association with risk factors by conducting a survey with adults in lists registered by family physicians. The study was conducted in the provinces of Marmara, one of Turkey's most industrialized and populous regions.

## 2. Materials and Methods

### 2.1. Study Design

The study was carried out in 10 provinces in the Marmara region: Balıkesir, Bilecik, Bursa, Edirne, Istanbul, Kırklareli, Kocaeli, Sakarya, Tekirdağ, and Yalova. Çanakkale Province was not included in the study because the physician in this province is no longer consulted there. A survey about hypertension was completed by adult patients (18 years or older) who had been diagnosed with having hypertension with a SBP ≥ 140 mmHg and/or DBP ≥ 90 mmHg (or already using an antihypertensive drug) by Family Health Center physicians between June 01, 2018, and November 30, 2018 in these provinces. The prevalence of hypertension in Turkey has been reported to be 31.8%. Considering this value, for a total population of 66694 registered patients over the age of 18 (combined number of patients registered at all of the Family Health Centers in the region), the necessary sample size for the study was calculated as 7407, with a significance level of 0.05 and an error of *d* = 0.01. After the sample size was calculated by the size-proportional stratification method, the percentage and number of samples per family physician were determined.

Written consent was obtained from each patient who agreed to participate. The 25-item questionnaire was administered to each participant by their family physician. The doctors read the questions out loud to the patients, and the patients provided a verbal response. The doctors documented the responses digitally, along with a digital copy of the questionnaire. The answers were recorded synchronously on the online web link provided by the Vademecum medication guide company. During the study period, the questionnaire was administered to a total of 2353 people with hypertension. The participants' BP readings were taken by a standard measurement method with automatic (arm-worn, electronic) BP measurement devices approved by the Turkish Society of Hypertension and Renal Diseases. Patients were asked about the duration and etiology of their hypertension, the medications they had used in the treatment of hypertension, and whether the medication they were using was their first treatment or if it was a modified treatment. Newly developed antihypertensives such as Mineralocorticoid Receptor Antagonists (MRAs), Aldosterone Synthase Inhibitors (ASIs), Aminopeptidase Inhibitors, Angiotensin Receptor-Neprilysin Inhibitors, Natriuretic Peptide Receptor Agonists (NPR-A), and Vasopeptidase Inhibitors were also asked about. The survey included questions about the participants' sociodemographic characteristics, such as their age, marital status, monthly income, and highest level of education. The survey also asked them to describe their alcohol consumption as “never use,” “social use,” or “continuous (regular) use.” They reported their smoking habits, diet, exercise status, comorbidities, hypertension risk factors, and the presence of conditions related to hypertension, such as retinopathy, nephropathy, and left ventricular hypertrophy. Those patients with organ involvement were also asked to provide retrospective glucose, urea, creatinine, glomerular filtration rate, and lipid parameters.

### 2.2. Statistical Analysis

SPSS 25.0 (IBM Corporation, Armonk, New York, United States) statistical software program was used in all calculations. Quantitative variables are expressed as mean ± SD (standard deviation), median, (minimum/maximum), percentile (25/75), and percentile (5/95), as shown in Tables 1 and 2. Categorical variables are given as *n* (%). It is more effective to use the central trend (mean and median) and central distribution measures (SD, min/max, and percentiles) for this type of study, as it provides detailed information for the general sample and population.

## 3. Results

Analysis indicated that the prevalence of hypertension in the Marmara region was 31.8%. Of the 2353 hypertensive patients who took part in the study, 1449 were women (61.6%) and 904 were men (38.4%). Among the respondents, 1555 (73.1%) had primary etiology and 572 (26.9) had secondary etiology. Regarding exercise, 1614 patients (68.6%) reported not exercising at all and 739 patients (31.4%) reported exercising regularly. Regarding diet, 1026 patients (43.9%) had not restricted their salt intake (Table 1).

The drug most commonly used by patients in this study to treat hypertension was an angiotensin converting enzyme (ACE) inhibitor (1264 patients, 32.7%). In contrast, only one patient was using a newly developed drug (0.1%). In terms of risk factors, 1032 (43.9%) of the patients were in the risky age group and 1134 (48.2%) had a family history of hypertension. There were 379 patients (16.1%) who self-reported as smokers. Diabetes mellitus was present in 692 patients (29.4%), hyperlipidemia in 538 patients (22.9%), retinopathy in 58 patients (5.1%), nephropathy in 66 patients (5.8%), and left ventricular hypertrophy in 51 patients (4.5%) (Table 2).

## 4. Discussion

In 2003, the PatenT study, a nationwide field survey of 4910 people, was conducted to determine the prevalence of hypertension in Turkey (PatenT: Prevalence, awareness, and treatment of hypertension in Turkey). The study results indicated the prevalence to be 31.8% of the adult population [15].

Our study was a similar cross-sectional field scan, and we found the prevalence of hypertension in the Marmara region to be 31.8%. Although this rate is considered high, it does indicate that hypertension prevalence has not increased since 2003. The practice of family medicine, which began in the last 10 years in Turkey, and the role of family physicians may have contributed to keeping the rate from increasing. Their success in curbing increasing rates of hypertension to the point where the rate has stabilized speaks to the effectiveness of the individual protection, diagnosis, treatment regulation, and strict follow-ups provided by the family physicians.

Another recent study reported the overall prevalence of hypertension as 32.96% [16]. In adults in the United States, the prevalence of hypertension was found to be 34.2% when using conventional threshold values of BP and 44.0% when using the newer recommendations (defining BP as elevated at 120–129/<80 mmHg and stage 1 hypertension at 130–139 and/or 80–89 mmHg) [17]. In the etiology of hypertension, primary or essential hypertension caused by environmental or genetic causes accounts for 90–95% of adult cases, while secondary hypertension caused by vascular, endocrine, and other causes constitute 2–10% of cases [18]. In our study, 1555 (73.1%) patients had primary etiology, and 572 (26.9%) had secondary etiology. This rate of secondary hypertension is much higher than the average 2–10% of cases. Therefore, further studies are needed to investigate the underlying conditions and determine the cause of the high secondary hypertension rate in Turkey. The fact that the Marmara region is the most industrialized and densely populated region of Turkey may be a factor related to the prevalence rates for hypertension among the patients in this study.

The majority of the participants with hypertension in this study did not exercise at all, and nearly half of them (43.9%) did not restrict their dietary intake of salt. These results emphasize the need for family physicians to apply a holistic approach to treating their patients who have hypertension. Physicians must work with these patients to increase their awareness of their condition and encourage them to make simple yet life-saving lifestyle changes to improve their health and reduce hypertension.

There are several different types or groups of antihypertensive medications that can effectively reduce BP. The Joint National Committee (JNC6) and (JNC7) recommend diuretics and beta blockers as the first choice in the treatment of uncomplicated hypertension [19, 20]. The drug most commonly prescribed to patients in our study is the one that affects the renin-angiotensin system. In contrast, the least used medication among the patients is from the newly developed drug group. One study reported that the most preferred drug group in the treatment of hypertension is an ACE inhibitor [21]. However, according to a study conducted in Brazil, the five most commonly used drugs were hydrochlorothiazide (a diuretic), losartan (an angiotensin receptor blocker), captopril (an ACE inhibitor), enalapril (an ACE inhibitor), and atenolol (a beta blocker), in descending order [22]. These results show that treatments may differ, as lifestyle factors, environmental factors, ethnicity, and the genetic composition of populations of different countries contribute to determining the most appropriate and most commonly used drugs for treating hypertension.

A study in China showed that five risk factors—increased age, male gender, city center work, cardiopulmonary insufficiency, and being overweight—were related to a higher prevalence of hypertension [23]. In our study, family history, age, diabetes mellitus, hyperlipidemia, and smoking were risk factors that were positively related to developing hypertension. In another study, metabolic syndrome, diabetes mellitus, and obesity were shown to be the conditions most commonly related to the development of hypertension [24]. These differing findings support the notion that cultural and environmental factors influence the prevalence of the condition.

A 2017 retrospective cohort study reported the incidence rate of target organ damage in hypertensive patients as 40.3%. The most common target organ damage was heart failure (11.6%), followed by ischemic heart disease (8.3%). The least common types of target organ damage were retinopathy (0.9%), atrial fibrillation (0.9%), and sexual dysfunction (0.67%) [25]. While 50% of patients with hypertension in our study did not know if they had organ involvement, 39.1% stated definitively that they did not. Others reported known related organ damage: 5.8% of the patients had nephropathy, 5.1% retinopathy, and 4.5% had left ventricular hypertrophy. These results show the need for awareness of organ involvement in patients with hypertension.

A study conducted in Canada reported that 67% of hypertension cases treated by family physicians in a 10-year period were well controlled through the physicians' care. The authors of the study suggest that a family physician may be the best suited clinician for detecting, evaluating, managing, and controlling hypertension. They also suggest that the family physician can be duly supported in providing care with help from secondary- and tertiary-care counselors, laymen, and the Ministry of Health in that country [26]. Similarly, based on the results of our study, we would like to emphasize that a coordinated approach involving family physicians and secondary and tertiary clinicians may be the most effective in the context of preventive medicine, diagnosis, treatment, and follow-up for hypertension. Additional support should be made available from specialist associations and health authorities.

### 4.1. Our Limitations

We were unable to attain the required sample size, in the current study. Since our study period was limited to six months and the working physician's resources were restricted, limited data could be collected. Since the selection of samples was randomized, even if only one-third of the required samples were collected, we believe that the sample size was large enough to not seriously affect the reliability of the results. Another limitation of this study was that the patients' examined lifestyle habits did not include their regular consumption of caffeinated beverages, particularly coffee or tea. It has been suggested that the consumption of caffeine correlates to BP and blood lipid levels. Future studies should include coffee and tea consumption as potential factors affecting BP. Another limitation is that the results are based on self-report survey responses rather than clinically measured data. There is always a risk of self-reported information lacking accuracy, either because participants are not entirely truthful or they may lack knowledge about some of the information requested. Therefore, even though many of our results are consistent with those from other studies in similar populations, they may not be entirely accurate. Additionally, it was not clear in our survey if the sodium intake reported by the patients refers only to the quantity they add to their food in cooking or at the table or if it represents their actual total sodium intake, including sodium naturally occurring in foods or added by manufacturers as a seasoning or as a preservative. Future studies addressing these issues may further clarify the relationship between these factors and the effective treatment of hypertension.

## 5. Conclusions

Our study results show that the prevalence of hypertension in Turkey's Marmara region is high, and, similar to the country in general, hypertension in Marmara is affected by different factors. However, although Marmara is the most crowded and industrialized region of the country, the prevalence of hypertension in that region is not higher than that for the country nationwide, suggesting that family medicine, put into practice in recent years in Turkey, has been successfully established. Individuals are benefitting from being closely monitored and cared for by their family physicians. Health policies that support family medicine and other clinicians should be developed to promote awareness of hypertension, its risk factors, and the severe complications that may arise. Further support for family medicine practitioners may positively affect public health.

## Figures and Tables

**Table 1:** Sociodemographic features, body measures, and duration of hypertension of the study participants.

		*n*			%	

*Province*						
	Balıkesir		32		1.4	
	Bilecik		24		1.0	
	Bursa		670		28.5	
	Edirne		17		0.7	
	İstanbul		813		34.6	
	Kırklareli		27		1.1	
	Kocaeli		508		21.6	
	Sakarya		68		2.9	
	Tekirdağ		149		6.3	
	Yalova		45		1.9	

*Gender*						
	Female		1.449		61.6	
	Male		904		38.4	

*Marital status*						
	Single		493		21.0	
	Married		1.860		79.0	

*Level of education*						
	Primary school or below		1.238		52.6	
	Secondary school		503		21.4	
	High school		309		13.1	
	University		303		12.9	

*Monthly income*						
	Over 10,000 TL		43		1.8	
	5,000 TL to 10,000 TL		221		9.4	
	Minimum wage or below		1.074		45.6	
	Minimum wage x 2		500		21.2	
	Minimum wage x 2 to 5,000 TL		515		21.9	

*Diet (daily salt intake)*						
	1 teaspoon		576		24.6	
	1 dessert spoon		563		24.1	
	1 tablespoon		173		7.4	
	No		1.026		43.9	

*Alcohol use*						
	Does not use		2.141		91.0	
	Social drinker		178		7.6	
	Continuous use		34		1.4	

*Smoking*						
	Quit		407		17.3	
	Uses		331		14.1	
	Does not use		1.615		68.6	

*Etiology*						
	Primary		1.555		73.1	
	Secondary		572		26.9	

*Exercise*						
	Yes		1.614		68.6	
	No		739		31.4	

	*n*	Mean ± SD	Median	(Min/max)	P. (25/75)	P. (5/95)
Height (cm)	2.305	163.00 ± 9.34	162.00	(140/205)	(156/170)	(15/179)
Weight (kg)	2.305	80.05 ± 14.90	79.00	(40/138)	(70/89)	(58/107)
BMI (kg/m^2^)	2.305	30.16 ± 5.31	29.65	(15.57/55.26)	(26.51/33.2)	(22.53/39.67)
Waist (cm)	2.303	100.71 ± 33.17	100.00	(10/1138)	(91/109)	(79/121)
Hip (cm)	2.303	112.78	108.00	(50/8893)	(101/116)	(92/130)
Duration of HT(months)	2.353	15.94 ± 15.53	11.00	(1/49)	(5/21)	(1/49)

P.: percentile, min: minimum, max: maximum, SD: standard deviation. BMI: body mass index; duration of HT: duration of hypertension.

**Table 2:** Medications used, risk factors, organ involvement, and laboratory findings for patients with hypertension.

		*n*			%	

*If taking medication; diuretic*						
	No		1.238		67.3	
	Yes		602		32.7	

*If taking medication; adrenergic antagonist*						
	No		1.459		79.3	
	Yes		381		20.7	

*If taking medication; renin-angiotensin system etkileyen ilaç*						
	No		576		31.3	
	Yes		1.264		68.7	

*If taking medication; vascular smooth muscle*						
	No		1.318		71.6	
	Yes		522		28.4	

*If taking medication; new drug being developed*						
	No		1.839		99.9	
	Yes		1		0.1	

*Risk factors; age (male 45 and female over 65)*						
	No		1.321		56.1	
	Yes		1.032		43.9	

*Risk factors; family history*						
	No		1.219		51.8	
	Yes		1.134		48.2	

*Risk factors; smoking*						
	No		1.974		83.9	
	Yes		379		16.1	

*Risk factors; diabetes mellitus*						
	No		1.661		70.6	
	Yes		692		29.4	

*Risk factors; hyperlipidemia*						
	No		1.815		77.1	
	Yes		538		22.9	

*Organ involvement; retinopathy*						
	No		1.076		94.9	
	Yes		58		5.1	

*Organ involvement; nephropathy*						
	No		1.068		94.2	
	Yes		66		5.8	

*Organ involvement; left ventricular hypertrophy*						
	No		1.083		95.5	
	Yes		51		4.5	

*Organ involvement; no*						
	No		772		68.1	
	Yes		362		31.9	

*Organ involvement; unknown*						
	No		510		45.0	
	Yes		624		55.0	

	*n*	Mean ± SD	Median	(Min/max)	P. (25/75)	P. (5/95)
Triglyceride (mg/dL)	1.868	157.67 ± 98.51	138.00	(1/1651)	(101/188)	(64/310)
Total chol (mg/dL)	1.883	210.59 ± 48.82	208.00	(12/800)	(179/239)	(137/290)
HDL chol (mg/dL)	1.860	51.47 ± 15.07	49.00	(4/173)	(42/58)	(32/80)
LDL chol (mg/dL)	1.800	130.54 ± 39.68	128.50	(12/326)	(104.5/152)	(69.5/199)
Glucose (mg/dL)	1.928	114.72 ± 39.17	103.00	(11/440)	(93/120)	(82/190)
Urea (mg/dL)	2.097	38.17 ± 28.61	31.00	(2.14/335.98)	(23/43)	(10/92.02)
Creatinine (mg/dL)	1.925	1.17 ± 04.26	0.81	(0.14/85)	(0.69/0.97)	(0.55/1.4)
GFR (ml/dk)	1.670	91.61 ± 38.61	90.80	(0.28/275.7)	(69/110.67)	(30.663/159.48)

P.: percentile, min: minimum, max: maximum, SD: standard deviation, total chol: total cholesterol, HDL chol: high-density lipoprotein cholesterol, LDL chol: low-density lipoprotein cholesterol, GFR: glomerular filtration rate.

## Data Availability

The data used to support the findings of this study are available from the corresponding author upon request.
